# Maltese cross: Starch artifact in oral cytology, divulged through polarized microscopy

**DOI:** 10.4103/0970-9371.66698

**Published:** 2010-01

**Authors:** Kiran Balasaheb Jadhav, Nidhi Gupta, Mujib BR Ahmed

**Affiliations:** Department of Oral and Maxillofacial Pathology, Bapuji Dental College and Hospital, Davangere, Karnataka, India

Sir,

An artifact (L.ars-art+factum-made) in histology means any nonnatural feature or structure accidentally introduced into something being observed or studied. Starch powder, a lubricant of surgical gloves, is well recognized as a common contaminant of cytological and histological specimens.[1]

Glove powder is chemically altered starch that includes donning powders, mold-release compounds and manufacturing debris. Cornstarch as a component of donning powder is the most common lubricant in gloves used for patient examination gloves.[2]

Although common, this artifact is potentially confusing in oral cytological specimens.[1] Recently, Pinto *et al*.[3] have reported starch artifacts in oral biopsy specimens. Starch powder is a well-documented iatrogenic cause for granulomatous lesions, both extraorally and intraorally.[1] In such situations, severe foreign body reactions may be seen, like starch granulomas. On the other hand, accidental starch granule contamination of biopsy tissues may occur during surgical removal or during specimen processing in the laboratory. In these cases, there is no inflammatory reaction associated with the starch granules.[3]

**Figure 1:**
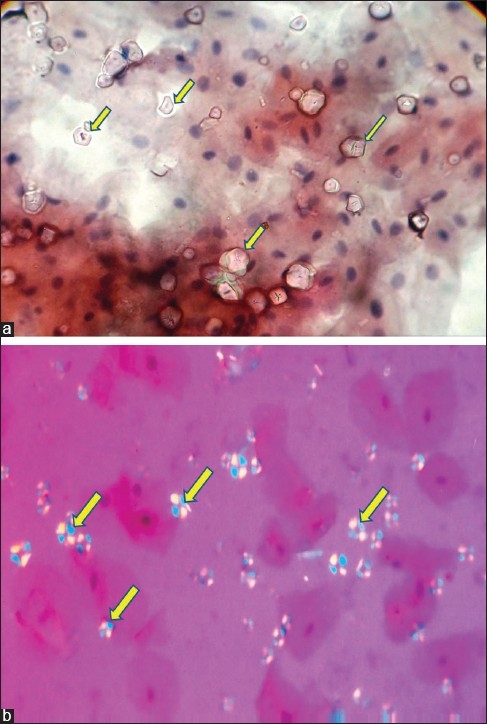
(a) PAP stained oral cytosmear under light microscope reveal presence of round to oval starch artifacts (arrow) with central dot or Y shaped structure (×200), (b) Oral cytosmear under polirized light reveal starch artifacsts (arrow) with “Maltese cross” birefringence pattern (×200)

A study was conducted to confirm the starch granules in cytosmears. The cytosmears were taken from the normal buccal mucosa of a healthy volunteer. Smears were taken without wearing gloves and again smears were repeated after palpating the buccal mucosa with gloves. These smears were stained with various staining methods, including rapid papanicolaou stain, hematoxylin and eosin stain, Masson trichrome stain, congo red stain and periodic acid schiff stain. These smears were then compared under a light microscope and a polarized microscope. The starch granules were absent in all smears from controls whereas they were found to be present in all the smears obtained following palpation of the same area with gloves [Figure 1].

Starch granules in cytologic smears appear as refractile, glassy, polygonal bodies, generally about 5–20 micrometer in diameter. They often exhibit a central dot or “Y”-shaped structure [Figure 1a]. The granules were PAS-positive and stained weakly green with Masson trichrome stain. Further evaluation under polarized light microscopy revealed the “Maltese cross” birefringence pattern, suggestive of starch granules [Figure 1b].

A central dark area, as in [Figure 1a], can be misinterpreted as a pyknotic nucleus or for cell undergoing mitosis under light microscope.[1] Moreover, Lovas *et al*.[1] have cited that these bodies might resemble epithelial cells. With scanning electron microscopy, starch granules appear as spherical, faceted balls, which can vary from 2.5 to 30 micrometer in diameter.[1] The same with scanning electron microscopy varied from 5 to 20 micrometer in diameter.[3] The Maltese cross under polarized light is characteristic, but not specific, for starch and can also be seen with some inorganic particles.[1]

Chlorinated natural latex rubber gloves are a common alternative to glove powder. Making use of synthetic polymer-coated gloves is yet another possibility.[2]

The purpose of this letter is to call the attention towards this artifact, which may be routinely found in a cytological smear. An adequate degree of consideration could significantly avoid erroneous conclusions.
